# Higher suicidality among primary care patients who have opioid use disorder and co-occurring depression and/or PTSD

**DOI:** 10.1186/s12875-026-03217-5

**Published:** 2026-02-24

**Authors:** Miriam Komaromy, Lisa S. Meredith, Maya Rabinowitz, Colleen McCullough, Lauren Kelly, Beth Ann Griffin, Sapna Mendon-Plasek, Katherine E. Watkins

**Affiliations:** 1 Grayken Center for Addiction, Boston Medical Center, Division of General Internal Medicine, Chobanian and Avedisian School of Medicine, Boston University, Boston, MA 02118 USA; 2 RAND, 1776 Main Street, Santa Monica, CA 90407-2138 USA; 3 RAND, 20 Park Plaza, Suite 910, Boston, MA 02116 USA; 4 RAND, 1200 South Hayes Street, Arlington, VA 22202-5050 USA

**Keywords:** Opioid use disorder, Depression, PTSD, Suicidality, Collaborative care, Safety net Federally Qualified Health Centers (FQHCs), Primary care, Integrating primary care and mental health

## Abstract

**Background:**

Depression and/or posttraumatic stress disorder (PTSD) commonly co-occur with opioid use disorder (OUD), and such co-occurring disorders (COD) are especially common among patients in primary care settings. Therefore, addressing the mental health needs of people with COD in primary care is essential.

**Methods:**

We compared the characteristics of three groups of patients who screened positive for OUD and depression and/or PTSD: (OUD + depression, OUD + PTSD, and OUD + depression + PTSD) and examined how membership in these groups was associated with suicidality and treatment. We analyzed cross-sectional baseline survey data from a larger study of collaborative care for COD. 797 adult patients were seen in 17 primary care clinics that provide care for diverse uninsured and underinsured patients in New Mexico and California. Our key independent variable was COD group. Dependent variables were suicidal ideation and behavior; and receipt of treatments for COD 30 days prior to baseline.

**Results:**

The patient group with all three conditions was larger than the other groups with only two disorders (OUD + PTSD (no depression) or OUD + depression (no PTSD). Patients with all three disorders reported more severe OUD, depression, and PTSD symptoms and higher probability of suicidality. Patients with PTSD (with or without depression) were more likely to report receiving medication for OUD and mental health counseling compared to those with no PTSD, and those with OUD, PTSD, and depression were more likely to report receiving medication for mental health problems than those with just two disorders.

**Conclusions:**

Higher probabilities of suicidality and of receiving mental health treatment among those with all three disorders point to the need for identifying these individuals in primary care.

**Trials registration:**

Clinicaltrials.gov, Identifiers, Registration Dates: NCT0455989; NCT04559893, September 8, 2020.

**Supplementary Information:**

The online version contains supplementary material available at 10.1186/s12875-026-03217-5.

## Background

Opioid use disorder (OUD) is a common condition with devastating consequences in the general U.S. adult population. As of 2023, the number of individuals who misuse opioids annually has climbed to 10 million. Opioids contribute to 70% of overdose deaths, resulting in almost 50,000 deaths annually [1, 2]. Beyond the risk of drug-related overdoses, recent research has begun to identify the connections between OUD and suicide. Studies suggest that people with OUD are 14 times more likely to die by suicide compared to the general population – a rate that is higher than that associated with any other substance [3].

The primary care setting offers a unique opportunity for prevention and early intervention to address misuse of opioids, as well as for treatment of OUD. Primary care providers (PCPs) are increasingly encouraged to address OUD [4]. However, provision of OUD care has been somewhat limited within primary care. In one study of six health systems, only 21% of patients with documented OUD received treatment with buprenorphine [5]. This is in part due to PCPs’ concern regarding their ability to address the mental health needs that frequently co-occur with OUD, including depression and Post Traumatic Stress Disorder (PTSD) [6].

Depression is highly prevalent in primary care patients across the U.S. adult population, with almost 30% of adults experiencing a depressive episode at least once in their lives [7]. Due to the prevalence of depression, screening is recommended in the primary care setting [8], and many providers report feeling comfortable initiating medication treatment or referring patients to specialized mental health care for depression [9]. While less prevalent than depression, PTSD is also relatively common in primary care settings, with a lifetime prevalence of 5–10% in U.S. adults [10]. Unlike depression, however, there has yet to be an established recommendation for PTSD screening in primary care practices, and PCPs are generally less familiar with diagnosis and treatment of PTSD compared with depression. Both depression and PTSD are associated with increased risk of suicide. For individuals with depression, the risk of suicide is approximately 8.6 times higher than the general population, and the risk of suicide for women with PTSD is 6.7 times higher than the general population [11]. For this reason, among others, it is essential to recognize, diagnose, and treat both disorders in an appropriate and timely manner.

Given the gaps in screening and treatment in primary care settings, it remains unclear how often PTSD and depression co-occur with OUD among patients who are treated in primary care settings, and how often all three disorders (depression, PTSD, and OUD) co-occur. The levels of suicidal ideation and behavior are also unknown in this population. The answers to these questions can inform the need for additional treatment resources in primary care settings and can help address the elevated care needs of patients with OUD and co-occurring depression and/or PTSD.

Earlier work by this team looked at the prevalence of co-occurring disorders (COD) in this population using a waiting room pilot study in four clinics in New Mexico. Data from this pilot identified that 4.5% of participants had OUD and among that subset, over half (2.4%) also had co-occurring depression and/or PTSD. In this study, we examined the frequency of co-occurrence between OUD and depression, PTSD, or both depression and PTSD. Using the full sample from primary care practices in New Mexico and California, we examined how the co-occurrence of these conditions correlates with suicidal ideation and behavior; as well as exploring how frequently treatment was provided for OUD and mental health disorders in patients who have COD.

## Methods

### Design, Setting, and Patients

Data for this paper were from a parent study called CLARO (Collaboration Leading to Addiction Treatment and Recovery from Other Stresses). CLARO is a pragmatic, randomized controlled trial in primary care clinics in New Mexico and California. The main study is testing whether patients with OUD and co-occurring depression and/or PTSD have better outcomes when they receive collaborative care compared with usual care. Patients were eligible if they met criteria for probable OUD and depression and/or PTSD based on interviewer administered self-report symptom severity scales (see Supplemental File). Additional details about the trial can be found in the protocol manuscripts [12, 13]. Here we report data on 797 patients from 17 clinics in New Mexico and California who completed the baseline survey by December 5, 2023. This population represented all (100%) of the CLARO patients enrolled in those clinics for the main study (n = 729) and for a small sub-study called CLARO + (*n* = 68).

### Assessments

Our key independent variable was co-occurring disorder group: OUD + depression, OUD + PTSD, and OUD + depression + PTSD. We examined five dependent variables. These include binary indicators of suicidal ideation in the 30 days prior to baseline and suicidal behavior ever (including preparatory acts and attempts) based on the Columbia Suicide Severity Rating Scale (C-SSRS) [14]. We measured receipt of treatment with three binary indicators of treatments received 30 days prior to baseline: (1) prescribed medication for OUD (MOUD), (2) counseling for depression or PTSD, and (3) prescribed medication for depression or PTSD.

Demographic characteristics included age (in years and by category: 18–30, 31–40, 41–50, 51 +); a binary indicator for female sex, and categories for race and ethnicity (White, non-Hispanic; Hispanic, all race; Other/more than one race, non-Hispanic), education (less than high school; high school or equivalent; some college or more), and marital status (never married; married/living with partner; widowed/divorced/separated). Clinical characteristics included a binary indicator of prior use of prescribed medication for OUD (MOUD) 30 days prior to baseline and measures of symptom severity for the co-occurring disorders 30 days prior to baseline. We measured OUD severity with the 7-item Patient-Reported Severity of Opioid Use Substance Misuse Short Form (PROMIS-SU-SF) [15]. We measured depression symptom severity with the 9-item Patient Health Questionnaire (PHQ-9) [16] and PTSD severity with the 20-item PTSD Checklist for DSM-V (PCL-5) [17]. All of these measures were rated on symptom frequency scales. Items were summed to create total scores in which higher scores indicate greater symptomatology. We also included categories for the different health systems (labeled systems 1–4).

### Statistical analysis

Missingness in our baseline data was generally very low (less than 5%). To clean up missing data, we first performed a series of logical imputations (e.g., participants who indicate not having suicidal thoughts were given an imputed score of no for the variable that captures intention to act on them) and calculated scaled sums for composite measures following best practices (e.g., participants who completed more than 50% of items of the PCL had non-missing items summed and then scaled up based on the number of missing items) [18]. Then for all other missing items, we use mean imputation within treatment assignment groups and health care system [19]. All data management and modelling was done in SAS 9.4 and bivariate comparisons and tables were done using Stata 17.

We first present bivariate data to describe the demographic and baseline clinical characteristics of the patients across the co-occurring disorder groups using analysis of variance (ANOVA) for continuous variables and chi-squared tests for categorical and binary measures. We then present adjusted logistic regression models for each dependent variable where the models control for the demographic characteristics listed above as well as type of health system. While we note that all reference groups for binary and categorical control covariates were selected to be the level with the largest sample size, we used OUD plus depression as the reference group for the primary independent variable to facilitate interpretation of this group to the other groups of interest in this study.

We report recycled predicted probabilities with 95% confidence intervals (CI) from each of these models to illustrate the size of the differences between the groups after adjustment [20]. Additionally, to reduce the probability of type I error with multiple testing of five outcomes, we performed a Benjamini–Hochberg correction with a false discovery rate (Q) = 0.05 on the joint F-tests to assess if findings were still statistically significant after adjustment for multiple testing [21].

## Results

The majority of patients in the sample had OUD and both depression and PTSD (59.1%), followed by those who had OUD and PTSD but no depression (22.4%), and those who had OUD and depression but no PTSD (18.4%). Table 1 shows baseline patient demographic and clinical characteristics for the full sample and by co-occurring disorder groups. Groups differed by age, with those in the OUD + PTSD group being younger compared with those in the OUD + depression group (*p* < 0.001). Otherwise, demographic characteristics did not differ by group. In terms of baseline clinical characteristics, OUD severity was lower for those in the OUD + PTSD group (*p* < 0.001) and, as expected, mental health symptom severity measures were higher for the groups with those disorders. The highest symptom severity scores were among those with all three disorders (*p* < 0.001 for both depression and PTSD).

**Table 1:** Baseline patient characteristics *,†,‡

**Characteristics**	**No. (%) of patients**				
	**All (*N* = 797)**	**OUD + Dep (*N* = 150)**	**OUD + PTSD (*N* = 176)**	**OUD + Dep + PTSD (*N* = 471)**	***P* value**
Demographic					
Age in years, mean (SD)	40.2 (11.9)	42.9 (14.1)	37.9 (11.0)	40.1 (11.2)	**< 0.001**
Age in years					**< 0.001**
18–30	177 (22.2)	29 (19.3)	46 (26.1)	102 (21.7)	N/A
31–40	298 (37.4)	50 (33.3)	77 (43.8)	171 (36.3)	N/A
41–50	158 (19.8)	22 (14.7)	27 (15.3)	109 (23.1)	N/A
51 +	164 (20.6)	49 (32.7)	26 (14.8)	89 (18.9)	N/A
Female	433 (54.3)	77 (51.3)	100 (56.8)	256 (54.4)	0.61
Race and Ethnicity					0.91
White, non-Hispanic	187 (23.5)	38 (25.3)	38 (21.6)	111 (23.6)	N/A
Hispanic, all race	543 (68.1)	98 (65.3)	124 (70.5)	321 (68.2)	N/A
Other/more than one race, non-Hispanic	67 (8.4)	14 (9.3)	14 (8.0)	39 (8.3)	N/A
Education					0.67
Less than high school	243 (30.5)	49 (32.7)	50 (28.4)	144 (30.6)	N/A
High school or equivalent	229 (28.7)	42 (28.0)	46 (26.1)	141 (29.9)	N/A
Some college or more	325 (40.8)	59 (39.3)	80 (45.5)	186 (39.5)	N/A
Marital status					0.39
Never married	288 (36.1)	53 (35.3)	70 (39.8)	165 (35.0)	N/A
Married/living with partner	294 (36.9)	62 (41.3)	64 (36.4)	168 (35.7)	N/A
Widowed/divorced/separated	215 (27.0)	35 (23.3)	42 (23.9)	138 (29.3)	N/A
Health system					0.83
System 1	381 (47.8)	70 (46.7)	82 (46.6)	229 (48.6)	N/A
System 2	35 (4.4)	9 (6.0)	8 (4.5)	18 (3.8)	N/A
System 3	321 (40.3)	57 (38.0)	75 (42.6)	189 (40.1)	N/A
System 4	60 (7.5)	14 (9.3)	11 (6.2)	35 (7.4)	N/A
Clinical					
Prior MOUD	721 (90.5)	129 (86.0)	164 (93.2)	428 (90.9)	0.08
OUD severity, mean (SD)	53.2 (8.4)	52.2 (8.9)	51.4 (7.5)	54.1 (8.3)	**< 0.001**
Depression symptom severity score, mean (SD); range: 0–24	13.8 (5.7)	13.8 (4.5)	7.0 (3.2)	16.4 (4.6)	**< 0.001**
PTSD symptom severity score, mean (SD); range: 0–80	38.1 (16.9)	27.4 (16.7)	30.6 (13.9)	43.3 (15.7)	**< 0.001**

*Data for this analysis were collected at baseline from January 8, 2021, through December 5, 2023

†P values are based on X2 for binary measures and Analysis of Variance (ANOVA) for continuous measures

‡OUD=opioid use disorder; Dep=Depression; PTSD=posttraumatic stress disorder

Adjusted logistic regression models (Table 2) indicate that having all three co-occurring disorders was associated with significantly higher odds of suicidal ideation (OR (odds ratio) = 1.71, 95% CI [1.12–2.61]), suicidal behavior (OR = 1.90, 95% CI [1.24–2.91]), receiving mental health counseling (OR = 1.77, 95% CI [1.16–2.71]), and receiving mental health medication (OR = 1.52, 95% CI [1.04–2.23]) compared to the group with OUD and depression. Having PTSD in addition to OUD, even without depression, is also associated with higher odds of receiving mental health counseling (OR = 1.73, 95% CI [1.05–2.83]) and receiving prescribed MOUD (OR = 1.74, 95% CI [1.00–3.01]) compared to having OUD with depression.

**Table 2:** Multivariable logistic regression results for suicide and treatment variables (*N* = 797)

**Dependent Variable**	Co-Occurring Disorder Group*,†,‡		
	**OUD + Dep *[Reference]***	**OUD + PTSD**	**OUD + Dep + PTSD**
Suicidal Ideation	N/A	0.70 (0.41–1.20)	1.71 (1.12–2.61)
Suicidal Behavior	N/A	1.04 (0.62–1.75)	1.90 (1.24–2.91)
Prescribed MOUD	N/A	1.74 (1.00–3.01)	1.54 (0.99–2.39)
Mental Health Counseling	N/A	1.73 (1.05–2.83)	1.77 (1.16–2.71)
Mental Health Medication	N/A	1.03 (0.66–1.63)	1.52 (1.04–2.23)

^*^Adjusted odds ratios (95% confidence intervals)

^†^Models are adjusted for covariates including demographic characteristics (age, sex, race/ethnicity, education, and marital status) and type of health system

^‡^*OUD* opioid use disorder, *Dep* Depression, *PTSD* posttraumatic stress disorder

**Fig. 1:**
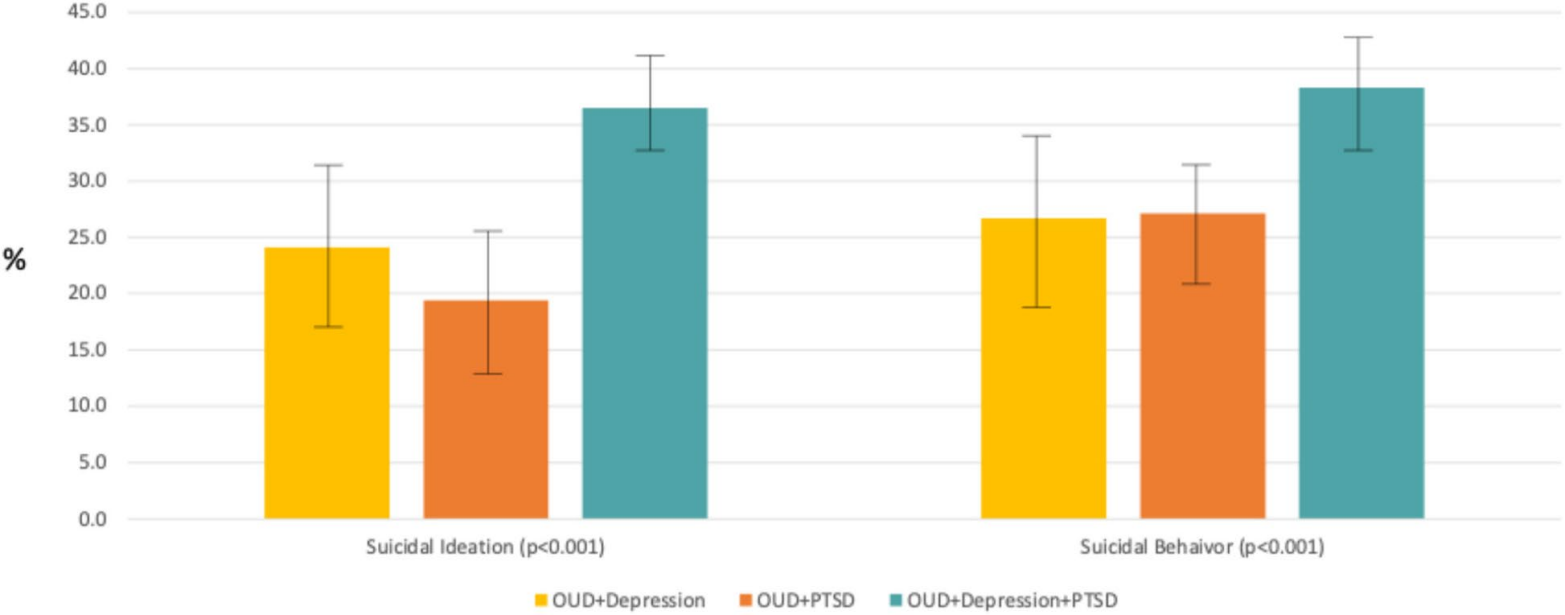
Adjusted probabilities of suicidal ideation and suicidal behavior by co-occurring disorder group*

**Fig. 2:**
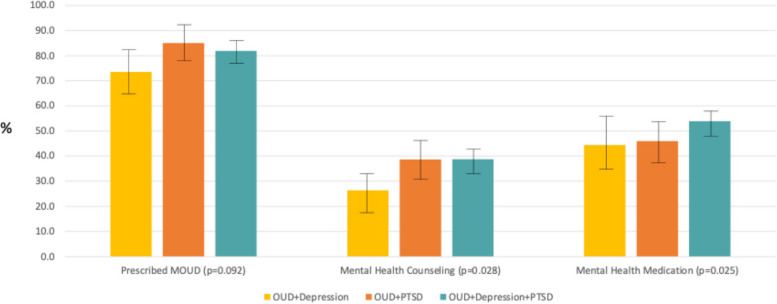
Adjusted probabilities of treatment received by co-occurring disorder group*

Figure 1 illustrates the predicted probabilities (along with error bars to depict the 95% confidence intervals) for suicidal ideation and suicidal behavior and Fig. 2 shows the probabilities for the receipt of treatment measures. As shown, the probability of both suicidal ideation and behaviors were significantly higher for the group with all 3 disorders (36.1%, 95% CI [31.8%−40.4%] for suicidal ideation and 38.1%, 95% CI [33.8%−42.4%] for suicidal behavior but only 19.1%−25.1% for the other groups for suicidal ideation and around 24.9%−25.7% for the other groups for suicidal behavior). For mental health treatments, the probability of MOUD were highest for the groups with PTSD (79.7–81.5%) versus only 72.4%, 95%CI [65.4%−79.4%]) in the group with OUD and depression. A similar pattern was seen for MH counseling with a probability of only 25.4%, 95% CI [18.3%−32.5%]) among the group with OUD and depression but probabilities around 36.7%−37.3% in the groups with PTSD. The probabilities of MH medicine use were not different between the groups and range from 42.2% to 52.3%. We note that the findings for suicidal ideation and behaviors were upheld after adjustment for multiple testing while the findings for the treatment variables were not, because the differences in suicide outcomes across the groups was larger in magnitude.

## Discussion

In this sample of primary care patients who have OUD plus a co-occurring mental health disorder, patients were more than twice as likely to have both depression and PTSD as they were to have either depression or PTSD alone. The combination of all three disorders was associated with substantially increased recent history of suicidal ideation and also lifetime suicidal behavior. Unfortunately, most patients reported receiving neither mental health counseling nor medication for these mental health disorders and the probability of reported receipt of counseling was lower than for reporting receipt of medications. This is consistent with data from the most recent National Surveys on Drug Use and Health [1] in which only 23% report any mental health treatment with prescription medincation, the most common among treatment options. It is unclear from our data to what extent the lack of receipt of treatment for mental health disorders was due to lack of availability of specialty mental health treatment, or instead due to patients not accessing treatment resources that were available, both of which are common in primary care settings. In all likelihood, it was partly due to each. This reinforces the importance of increasing the availability of mental health services in primary care settings, and of identifying and facilitating patients with co-occurring SUD and mental health disorders to access the services.

Our findings suggest that primary care providers may be willing to prescribe MOUD for the treatment of OUD but are much less likely to refer patients to counseling for either OUD or co-occurring disorders. However, the mental health disorders that commonly co-occur with OUD often present as complex clinical profiles, and these patients are at high risk for psychological distress (suicidal ideation) and attempts to harm themselves. This level of complexity is difficult to address in a brief primary care visit, and PCPs rarely feel that they have adequate knowledge or resources to prepare them to deal with these multiple diagnoses [22]. PCPs should therefore refer these people to behavioral health services because they need counseling to address their complex co-occurring problems. Nonetheless, people who have OUD and common mental health disorders such as depression and PTSD are more likely to be seen in primary care settings than in other outpatient healthcare treatment settings [23, 24].

Although it is not realistic to expect primary care teams to provide comprehensive treatment for these disorders on their own, it is essential that detection of these disorders occurs at the primary care level so that patients have an opportunity to access treatment. The USPSTF recommends screening for OUD (as part of general screening for SUD in all adults) [25]. Even if primary care clinics are unable to provide comprehensive treatment for patients who have OUD and common mental health disorders, clinics can enhance their ability to identify these patients by adding PTSD screening for patients with OUD [26, 27]. Implementing PTSD screening in patients with OUD, particularly with an instrument that has been validated in patients who have co-occurring SUDs, can help to identify this group of patients.

Although most primary care practices are unlikely to undertake treatment of OUD and co-occurring mental health diagnoses on their own, they may operate in areas where access to specialty care from mental health therapists is limited [28], particularly for low-income patients, and primary care practices may therefore provide the only treatment that is available. There are at least three promising strategies to help enhance the capacity to address these problems in primary care. The first is the primary care health integration model [29], in which a licensed behavioral health professional is integrated into primary care team to provide on-site, routine, and population-based services. Potential benefits include improved patient outcomes, increased access to care; and more efficient, coordinated, care.

A second strategy is offered through the Extension for Community Healthcare Outcomes (ECHO) model [30–34], which provides a virtual learning community in which primary care teams can learn about how to treat SUDs and co-occurring mental health diagnoses and can present de-identified patient cases and receive coaching from experts.

A third potential model is collaborative care [35, 36]. The Collaborative Care Model (CoCM) may be helpful for patients with co-occurring OUD and mental illness, given the potential to bill for the extra care provided to address behavioral health issues. In this model, the primary care team is augmented by a care manager who works to ensure that a panel of patients is receiving high quality care, and the primary care team is supported through virtual consultation with a specialist in mental health and SUD care. This model is eligible for reimbursement [37] through Collaborative Care billing codes [38]. As of 2022 [39, 40], approximately one third of states reimbursed for CoCM codes. However, implementation [41–43] of these codes may also be challenging, and uptake has been slow, thereby hindering primary care practices to access this import source of support.

While this study made important contributions to understanding the increased risk of suicidal ideation and behavior among those with co-occurring disorders, there are some limitations to note. Our analyses were cross-sectional, and we cannot establish any causal relationships. In addition, we explored patient characteristics across the three groups of patients in low-income and predominately Hispanic clinics in New Mexico and California – who experienced high stigma around mental health [44] disorders – from primary care settings. As such, we cannot know whether our findings would hold in other states and settings. It is also possible that the aforementioned promising models of care, may not be as effective or cost-effective for complex patient populations as for those with more typical mental illness (e.g., mild-to-moderate depression where the evidence is strong) [29]. Thus, it is unclear whether redirecting primary care resources to address this population would be the best use of resources beyond standard specialty care. Approximately 80% of CLARO study participants reported that they had taken MOUD in the 30 days prior to enrollment, although some of this exposure undoubtedly represents people using MOUD that has not been prescribed for them. These levels of receipt of MOUD treatment are much higher than proportions of patients with OUD who are prescribed MOUD that are reported in the literature [5, 45, 46]. Possible reasons for this include local factors in New Mexico, where most of the study enrollment took place. New Mexico treatment providers have elevated awareness of the toll of overdose deaths, since New Mexico’s drug overdose death rate has been one of the highest in the nation for most of the last two decades [47]. Another factor that has likely increased MOUD prescribing in New Mexico is exposure to Project ECHO learning networks focused on increasing treatment of OUD. ECHO participation is associated with increased rates of MOUD prescribing [48, 49], and project ECHO has been providing this education in New Mexico since 2005 [34].

Additionally, data were all based on self-report which may be subject to biases such as responding in a socially desirable manner. Lastly, we were unable to include a control group without OUD or with OUD but without co-occurring mental health disorders to allow for a fuller understanding of what the rates of suicidality might be in these groups of individuals. Future work should aim to more fully compare such groups to understand the role of severity of mental health disorders in these groups.

## Conclusions

Depression, PTSD, and OUD co-occur more commonly than OUD plus depression or PTSD alone. We observed very high probabilities of reported suicidal ideation and behavior among patients with OUD and co-occurring depression or PTSD, and especially for those with depression plus PTSD together. However, fewer than half of patients who were seen in primary care settings with these disorders report receiving treatment. Screening and intervention for depression, PTSD and suicidality are essential in patients with OUD.

## Supplementary Material

Supplementary Material 1

## Data Availability

The authors declare that the data supporting the findings of this study are available from the corresponding author upon request.

## Abbreviations

ANOVA: Analysis of variance
CI: Confidence interval
CoCM: Collaborative care management
COD: Co-occurring disorders
DSM-V: Diagnostic and Statistical Manual, version five
MOUD: Medication for opioid use disorder
OR: Odds ratio
OUD: Opioid use disorder
PCL-5: Five item PTSD checklist
PCP: Primary care provider
PHQ-9: Nine item patient health questionnaire
PROMIS-SU-SF: Patient-Reported Severity of Opioid Use Substance Misuse Short Form
PTSD: Posttraumatic stress disorder
