# Supplementary material for: Higher suicidality among primary care patients who have opioid use disorder and co-occurring depression and/or PTSD
Source: BMC Prim Care. 2026 Feb 24;27:113. doi: 10.1186/s12875-026-03217-5 (PMC13040686; doi:10.1186/s12875-026-03217-5)
Supplement: Supplementary file 1 — Supplementary Material 1 [file 12875_2026_3217_MOESM1_ESM.docx]

**CLARO Eligibility Screeners**

**myTAPS Questions**

**We are going to ask you some questions about using different drugs. All of your answers are confidential, and we will not share this information with anyone.**

1. In the past 90 days, have you taken any Buprenorphine (Suboxone, Zubsolv, Subutex, Sublocade), Methadone, or Injectable Naltrexone (Vivitrol) for problems with heroin or pain pills? Sometimes people get these medicines from a prescription and sometimes they get them on the street.

□ No □ Yes

☐ DON’T KNOW

☐ REFUSE

If YES 🡪 skip myTAPS (Q2-Q7) and go to PHQ-8

If NO/DON’T KNOW/REFUSE 🡪 continue with myTAPS (Q2-Q7)

2. In the PAST 3 MONTHS, did you take any prescription pain pills, even just once (for example: Oxycodone, Percocet, Vicodin)?

□ No □ Yes

If NO 🡪 skip to Question 5

☐ DON’T KNOW

☐ REFUSE

(2a) In the PAST THREE MONTHS, did you take any prescription pain pills that were NOT prescribed for you?

□ No □ Yes

If YES 🡪 skip to Question 3

☐ DON’T KNOW

☐ REFUSE

(2b) In the PAST THREE MONTHS, have you ever taken more prescription pain pills than your provider told you to?

□ No □ Yes

If NO 🡪 skip to Question 5

☐ DON’T KNOW

☐ REFUSE

3. In the PAST 3 MONTHS, have you tried and failed to control, cut down or stop using prescription pain pills?

□ No □ Yes

☐ DON’T KNOW

☐ REFUSE

4. In the PAST 3 MONTHS, has anyone told you they were worried about your use of prescription pain pills?

□ No □ Yes

☐ DON’T KNOW

☐ REFUSE

5. In the PAST 3 MONTHS, did you use heroin?

□ No □ Yes

If NO to Question 4 🡪 skip Q6 and Q7

☐ DON’T KNOW

☐ REFUSE

6. In the PAST 3 MONTHS, have you tried and failed to control, cut down or stop using heroin?

□ No □ Yes

☐ DON’T KNOW

☐ REFUSE

7. In the PAST 3 MONTHS, has anyone told you they were worried about your use of heroin?

□ No □ Yes

☐ DON’T KNOW

☐ REFUSE

If Q1 = NO/DON’T KNOW/REFUSE **AND** myTAPS = NO/DON’T KNOW/REFUSE for Q3, Q4, Q6, AND Q7 🡪 end survey

**Citation: Adam A, Schwartz RP, Wu L-T, Subramaniam G, Laska E, Sharma G, Mili S, McNeely J. Electronic self-administered screening for substance use in adult primary care patients: Feasibility and acceptability of the tobacco, alcohol, prescription medication, and other substance use (myTAPS) screening tool. *Addiction Science & Clinical Practice.* 2019;14(1):39. doi: 10.1186/s13722-019-0167-z.**

**PHQ-8 Questions**

**The following questions will ask how often you have been bothered by different problems.**

**Over the LAST TWO WEEKS, how often have you been bothered by any of the following problems?**

|  | **Not at all** | **Several days** | **More than half the days** | **Nearly every day** | **DON’T KNOW** | **REFUSE** |
| --- | --- | --- | --- | --- | --- | --- |
| **1. Little interest or pleasure in doing things** | ☐ | ☐ | ☐ | ☐ | ☐ | ☐ |
| **2. Feeling down, depressed, or hopeless** | ☐ | ☐ | ☐ | ☐ | ☐ | ☐ |
| **3. Trouble falling or staying asleep, or sleeping too much** | ☐ | ☐ | ☐ | ☐ | ☐ | ☐ |
| **4. Feeling tired or having little energy** | ☐ | ☐ | ☐ | ☐ | ☐ | ☐ |
| **5. Poor appetite or overeating** | ☐ | ☐ | ☐ | ☐ | ☐ | ☐ |
| **6. Feeling bad about yourself – or that you are a failure or have let yourself or your family down** | ☐ | ☐ | ☐ | ☐ | ☐ | ☐ |
| **7. Trouble concentrating on things, such as reading the newspaper or watching television** | ☐ | ☐ | ☐ | ☐ | ☐ | ☐ |
| **8. Moving or speaking so slowly that other people could have noticed. Or the opposite – being so fidgety or restless that you have been moving around a lot more than usual** | ☐ | ☐ | ☐ | ☐ | ☐ | ☐ |

**Citation: Spitzer RL, Kroenke K, Williams JB. Validation and utility of a self-report version of PRIME-MD: The PHQ primary care study. Primary care evaluation of mental disorders. Patient health questionnaire. *JAMA.* 1999;282(18):1737-1744. doi: 10.1001/jama.282.18.1737.**

**PC-PTSD-5 Questions**

**Sometimes things happen to people that are unusually or especially frightening, horrible, or traumatic. For example:**

**• a serious accident or fire**

**• a physical or sexual assault or abuse**

**• an earthquake or flood**

**• a war**

**• seeing someone be killed or seriously injured**

**• having a loved one die through homicide or suicide.**

Have you ever experienced this kind of event? YES NO

☐ DON’T KNOW

☐ REFUSE

If NO 🡪 skip to end of survey

If DON’T KNOW/REFUSE 🡪 end survey

In the PAST MONTH, have you…

1. had nightmares about the event(s) or thought about the event(s) when you did not want to?

YES (1) NO (0)

☐ DON’T KNOW

☐ REFUSE

2. tried hard not to think about the event(s) or went out of your way to avoid situations that reminded you of the event(s)?

YES (1) NO (0)

☐ DON’T KNOW

☐ REFUSE

3. been constantly on guard, watchful, or easily startled?

YES (1) NO (0)

☐ DON’T KNOW

☐ REFUSE

4. felt numb or detached from people, activities, or your surroundings?

YES (1) NO (0)

☐ DON’T KNOW

☐ REFUSE

5. felt guilty or unable to stop blaming yourself or others for the event(s) or any problems the event(s) may have caused?

YES (1) NO (0)

☐ DON’T KNOW

☐ REFUSE

**Citation: Prins A, Bovin MJ, Smolenski DJ, Marx BP, Kimerling R, Jenkins-Guarnieri MA, Kaloupek DG, Schnurr PP, Kaiser AP, Leyva YE. The primary care PTSD screen for DSM-5 (PC-PTSD-5): Development and evaluation within a veteran primary care sample. *Journal of General Internal Medicine.* 2016;31(10):1206-1211. doi:**
